# Effectiveness and Safety of Mefenamic Acid Oral Suspension in Pediatric Practice for Febrile Illness: An Observational Study (EASE-O-MAPP Study)

**DOI:** 10.7759/cureus.100469

**Published:** 2025-12-31

**Authors:** Vasant Khalatkar, Prabhu N Kasture, Devesh Kumar

**Affiliations:** 1 Pediatrics, Khalatkar Hospital, Nagpur, IND; 2 Medical Services and Pharmacovigilance, Blue Cross Laboratories Pvt. Ltd., Mumbai, IND; 3 Clinical Operations, IR Innovate Research Pvt. Ltd., Noida, IND

**Keywords:** antipyretics, children, febrile illness, fever, mefenamic acid, nlrp3, nsaid, pediatrics

## Abstract

Introduction

Fever and associated pain are among the most common pediatric symptoms. If not managed promptly, they can lead to discomfort, irritability, and reduced well-being in the child, as well as increased anxiety among caregivers. Mefenamic acid is a nonsteroidal anti-inflammatory drug (NSAID) that exhibits antipyretic, analgesic, and anti-inflammatory properties, making it a suitable option for symptomatic management. Pediatric oral suspension allows for weight-based dosing and convenient administration of mefenamic acid in children. This study aims to evaluate the real-world effectiveness and safety of mefenamic acid oral suspension in the treatment of fever and associated symptoms in pediatric patients.

Methods

This single-center, prospective, open-label, investigator-initiated, observational study was conducted between December 30, 2024, and April 4, 2025. The study included 50 pediatric subjects (six months to <18 years) with fever (>99.5°F). Mefenamic acid oral suspension was administered at a dose of 5 mg/kg three times daily for up to three days, or until normothermia was achieved, whichever occurred earlier. Efficacy was evaluated based on the degree of fever reduction and the time required to achieve sustained normothermia. Safety assessment included laboratory and clinical evaluations, such as blood tests, urinalysis, fecal occult blood testing (FOBT), completion of a gastrointestinal (GI) symptom questionnaire, and monitoring for adverse events.

Results

A total of 50 participants completed the study. The major causes of fever were upper respiratory tract infection (72%) and lower respiratory tract infection (24%). A significant decrease in mean body temperature was observed from baseline (102.36 ± 0.78°F) to the end of treatment on day 3 (98.61 ± 0.69°F; p < 0.0001). The mean time to achieve normothermia after the first dose was 1.99 ± 0.47 hours, while sustained normothermia was maintained for an average of 61.92 ± 11.52 hours. No treatment-related adverse events or clinically significant laboratory abnormalities were observed during the study period.

Conclusion

Mefenamic acid oral suspension demonstrates a rapid and sustained antipyretic effect in pediatric patients, producing significant temperature reduction without affecting hematological or biochemical parameters. These findings confirm its efficacy, safety, and good GI tolerability in the management of febrile illnesses among children.

## Introduction

Fever associated with pain is a common pediatric symptom arising from infections or systemic inflammation and involves a complex immune-neuroendocrine response. Key mechanisms include the release of pyrogenic cytokines (IL-1β, IL-6, and TNF-α) through activation of the NLRP3 inflammasome, which, in turn, promotes prostaglandin E₂ (PGE₂) synthesis via COX pathways, resetting the hypothalamic set point. Fever generation occurs through both humoral (cytokine-driven PGE₂ production) and neuronal (vagal afferent signaling) pathways, working in concert to elevate body temperature and mount a coordinated febrile response [1].

Current pediatric fever management guidelines prioritize the overall comfort of the child rather than routine temperature reduction, recommending antipyretic use when fever is accompanied by significant distress or discomfort and emphasizing weight-based, safe dosing [2].

Mefenamic acid, a fenamate-class nonsteroidal anti-inflammatory drug (NSAID), is widely used in pediatric populations for its antipyretic, anti-inflammatory, and analgesic properties [3]. Its unique antipyretic action involves preferential COX-2 inhibition, EP receptor blockade, and NLRP3 inflammasome suppression, distinguishing it from other NSAIDs [4]. Distinctively, mefenamic acid not only suppresses PGE₂ synthesis but also blocks PGE₂ binding at EP receptors, providing receptor-level antagonism. The dual mechanism - COX inhibition and EP receptor blockade - enhances its antipyretic potency. Additionally, mefenamic acid inhibits NLRP3 inflammasome activation, preventing IL-1β release and subsequent stimulation of hypothalamic fever centers [5,6].

Recent comparative clinical evidence also supports these mechanistic advantages, showing that mefenamic acid delivers antipyretic efficacy comparable to high-dose paracetamol with a longer duration of fever control, highlighting its potential superiority in sustaining antipyresis [7]. These mechanistic insights highlight the clinical relevance of mefenamic acid oral suspension in managing pediatric fever.

Despite longstanding clinical use, there is limited real-world evidence on the effectiveness and safety of mefenamic acid oral suspension in routine pediatric practice. This gap prompted the present observational EASE-O-MAPP study. Accordingly, the study was designed with the objectives of evaluating the effectiveness of mefenamic acid oral suspension at an optimal dosing of 5 mg/kg per dose in reducing fever among pediatric and adolescent patients with febrile illness and assessing its safety and tolerability at this standardized dose. The study endpoints included monitoring treatment-related and serious adverse events throughout the study period using clinical assessments, laboratory parameters, and a gastrointestinal (GI) tolerability questionnaire, while effectiveness was evaluated by reduction in body temperature and the time required to achieve sustained normothermia - defined as a body temperature ≤99.5°F maintained for at least 24 hours.

## Materials and methods

Study design and ethical approval

This single-center, prospective, open-label, investigator-initiated, observational study was conducted at the Pediatric Outpatient Department of Khalatkar Hospital, Nagpur, India. Patients were recruited consecutively between December 30, 2024, and April 5, 2025. The objective was to evaluate the efficacy and safety of mefenamic acid oral suspension in febrile pediatric patients.

Ethical approval was obtained from the Institutional Ethics Committee of Khalatkar Hospital (Reference No. IIS-MEF/CT/2024/01; dated December 20, 2024), and the study was registered with the Clinical Trial Registry of India (CTRI/2025/01/078895). It was conducted in accordance with the Declaration of Helsinki, ICH-GCP guidelines, and NDCT Rules 2019. Informed consent was obtained from all parents or legal guardians before enrolment.

Study population

Children between six months and under 18 years of age presenting with febrile illness (temperature range: 100.5-104°F) were enrolled in the study. Exclusion criteria included recent use of NSAIDs or paracetamol, known hypersensitivity to mefenamic acid or other NSAIDs, hepatic or renal dysfunction, dehydration, or any serious comorbid medical condition.

Study treatment

Participants received an oral suspension of mefenamic acid at a dose of 5 mg/kg three times daily for up to three days or until normothermia was achieved, whichever occurred earlier. The specific formulation used in this study was Mefenamic Acid Oral Suspension (Meftal-P®, Blue Cross Laboratories Pvt. Ltd., Mumbai, India). Each participant was followed for a total of seven days, encompassing both treatment and post-treatment observation periods.

If fever or associated symptoms remained uncontrolled after two doses of the study medication, or if the child remained distressed, rescue treatment with standard clinical care could be administered at the investigator’s discretion, followed, if necessary, by any further escalation of therapy.

Study assessments

Clinical evaluations were performed on Day 1 (baseline), Day 3, and Day 7, including vital signs, physical examination, and temperature monitoring. Efficacy was assessed by percentage reduction in temperature, time to sustained normothermia, and number of fever-free time points. Sustained normothermia was defined as a body temperature ≤99.5°F maintained for ≥24 hours, consistent with pediatric fever resolution criteria [8,9]. Body temperature was measured with digital axillary thermometers with an accuracy of ±0.1°C. Caregivers received training on correct axillary placement and were instructed to keep the arm in position for the specified measurement time. Temperature records were maintained in structured diaries and reviewed at each study visit. Primary temperature outcomes were assessed over the first 72 hours, corresponding to the acute febrile phase, during which antipyretic efficacy is expected.

Safety assessments included laboratory tests, fecal occult blood test (FOBT), and a GI tolerability questionnaire (Appendix 1). Treatment compliance was checked via diary review and medication return.

Sample size calculation

This sample size (n = 50) was chosen pragmatically for an observational exploratory study and not based on a formal hypothesis-testing framework.

Statistical analysis

Descriptive statistics were used to summarize the study data. Continuous variables were expressed as mean ± standard deviation (SD), while categorical variables were summarized as counts and percentages. Changes from baseline in continuous variables were evaluated using paired t-tests or appropriate non-parametric alternatives when normality assumptions were not met. Efficacy was evaluated in the Full Analysis Set (FAS), which included all participants who received at least one dose of the study drug and had at least one post-baseline assessment, and in the Per-Protocol Set (PPS), which included participants who completed the study without major protocol violations. Safety and tolerability were assessed in the Safety Analysis Set (SAF), consisting of all participants who received at least one dose of the study medication. Missing data were managed using multiple imputation or the last observation carried forward (LOCF) approach, as deemed appropriate. All statistical analyses were performed using R 4.4.1 software (The R Foundation for Statistical Computing, Vienna, Austria), and the level of statistical significance was set at p < 0.05 for all analyses.

## Results

Demographic and baseline characteristics

**Figure 1:**
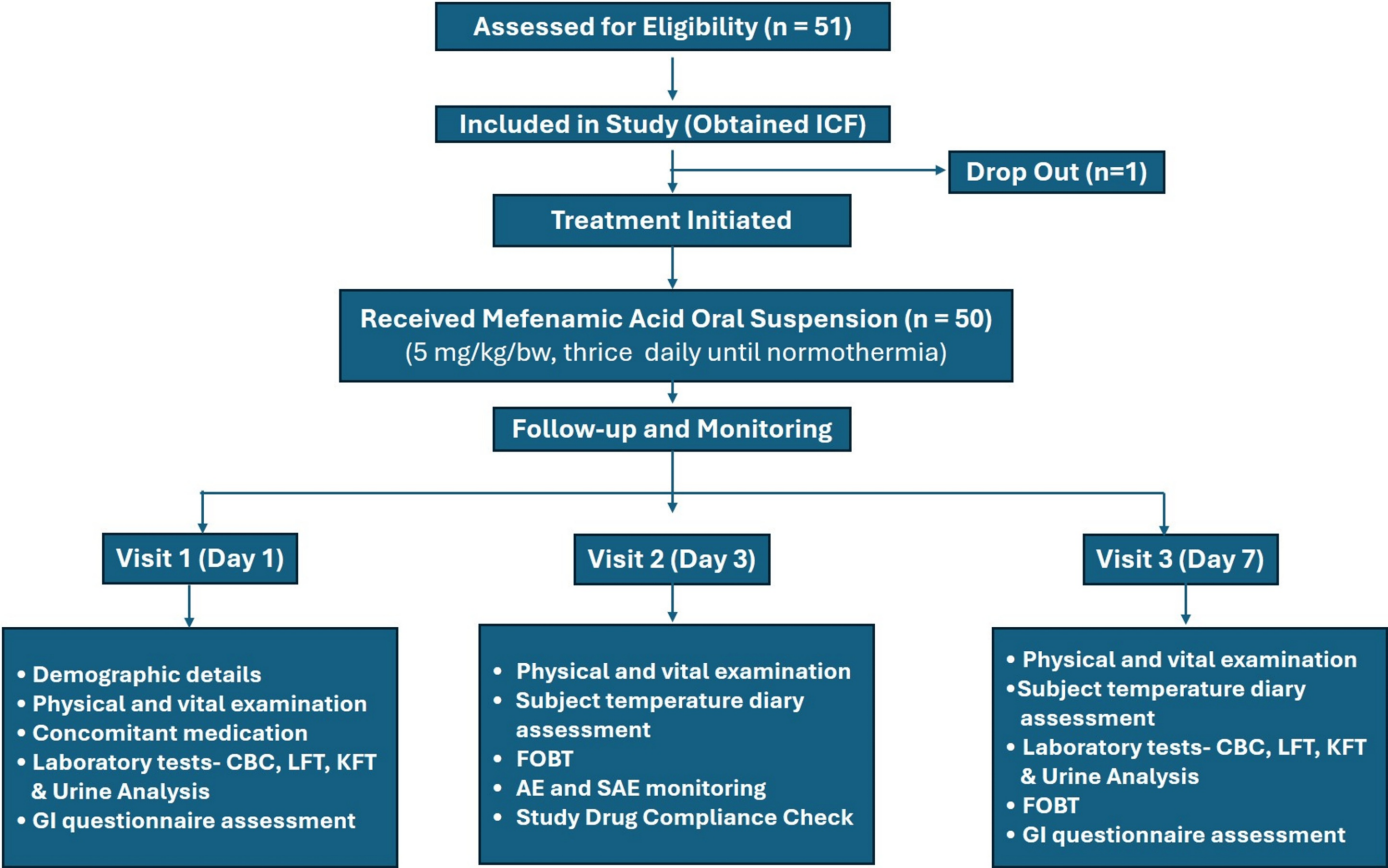
Participant Flow Diagram AE: Adverse Event; CBC: Complete Blood Count; FOBT: Fecal Occult Blood Test; GI: Gastrointestinal; ICF: Informed Consent Form; KFT: Kidney Function Test; LFT: Liver Function Test; mg/kg/bw: Milligram per Kilogram of Body Weight; n: Number of Subjects/Participants; SAE: Serious Adverse Event

A total of 51 children were enrolled in the study, of whom one participant was lost to follow-up, resulting in 50 evaluable participants in the per-protocol analysis (Figure 1).

The study included children aged six months to under 18 years, with 72% (n = 36) in the younger age group (six months to five years) and 28% (n = 14) in the older group (6-18 years). The mean height was 90.28 ± 14.85 cm and 136.93 ± 13.27 cm for the younger and older groups, respectively. Mean weights were 11.59 ± 2.76 kg and 31.59 ± 9.06 kg, with corresponding BMI values of 14.67 ± 3.60 kg/m² and 16.47 ± 2.65 kg/m². At baseline, the mean body temperature was 102.36 ± 0.78°F.

The causes of fever were predominantly viral upper respiratory tract infections, with 36 subjects (72%) presenting with nasal/throat congestion, cough, and cold, consistent with the common cold, pharyngitis, viral tonsillitis, or acute sinusitis. Lower respiratory tract involvement was identified in 12 subjects (24%) as viral bronchiolitis or respiratory infection. Additionally, urinary tract infection was noted in two subjects (4%).

Common concomitant medications included multivitamins (94%, n = 47), antibiotics (62%, n = 31), expectorants (52%, n = 26), bronchodilators/mucolytics (28%, n = 14), antihistamines (16%, n = 8), and inhalational corticosteroids (14%, n = 7). Less commonly used concomitant agents were antioxidants, antiemetics, and probiotics - all prescribed based on clinical judgment.

Assessment of efficacy

Efficacy was evaluated as the mean reduction in fever for three consecutive days, the time to achieve sustained normothermia, and the time to fever-free time points. Evaluation of patients for reduction in body temperature at three time points across three consecutive days is summarized in Table 1.

**Table 1:** Reduction in Fever (n = 50) Values expressed as mean ± SD. *A t-test p-value < 0.05 was considered statistically significant.

Time-Points	Mean Body Temperature (°F)			Mean Reduction From Baseline	t-value	p-value
	Day 1	Day 2	Day 3			
Morning	-	101.19 ± 0.79	99.56 ± 0.77	-0.67 ± 0.37	-24.27	<0.0001*
Noon	102.41 ± 0.77	100.70 ± 0.68	99.14 ± 0.71	-2.27 ± 0.72	-27.85	
Evening	101.72 ± 0.69	100.09 ± 0.68	98.61 ± 0.69	-3.71 ± 0.84	-31.23	

All participants received their first dose on the afternoon of Day 1, with a mean reduction of -0.67 ± 0.37 in body temperature by evening. Day 2 showed further decrease in body temperature, with cumulative reductions of -1.19%, -1.66%, and -2.27%, respectively. By the third day of treatment, all participants showed a marked decline in body temperature, with most approaching normal levels. The mean morning temperature was 99.56 ± 0.77°F, with 25 of 50 children afebrile. By midday, the mean temperature further decreased to 99.14 ± 0.71°F, and 40 participants were afebrile. By evening, the mean temperature reached 98.61 ± 0.69°F, with 45 of 50 children being afebrile. This corresponded to a mean reduction of -3.71 ± 0.84°F from baseline, indicating a consistent and sustained antipyretic effect. Complete normothermia was observed in all participants by Day 4, following nine doses, and they remained afebrile during the follow-up period.

**Figure 2:**
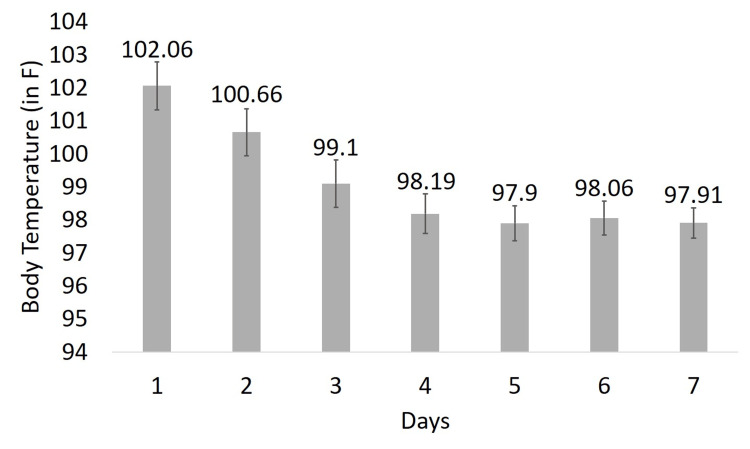
Mean Body Temperature Over Seven Days Error bars represent standard deviation.

The mean daily body temperature demonstrated a steady decline throughout the treatment period. The mean body temperature declined from approximately 102.06°F on Day 1 to 99.10°F by Day 3, representing a statistically significant reduction (p < 0.0001) with normothermia sustained through Day 7 of the study. This finding confirms the antipyretic efficacy of mefenamic acid oral suspension (Figure 2).

Fever-free timepoints (temperature ≤ 99.5°F) increased steadily during the study. From a baseline mean of 0.00 ± 0.00, values rose to 0.22 ± 0.58 on Day 2 and 2.20 ± 0.98 on Day 3. By Day 4, all subjects achieved and maintained complete normothermia (3.00 ± 0.00) through Day 7, indicating effective fever resolution. The mean time to achieve normal body temperature after the first dose of mefenamic acid oral suspension was 1.99 ± 0.47 hours, and the average time required to achieve and maintain a body temperature ≤ 99.5°F for 24 continuous hours was 61.44 ± 10.17 hours.

Changes in vital parameters

Vital parameters were monitored at baseline, mid-treatment, and the end of the study to assess the physiological response and safety profile of the intervention. Systolic blood pressure remained stable (77.06 ± 13.75 mmHg at baseline to 77.66 ± 16.49 mmHg on Day 7). Diastolic pressure showed a slight increase (from 49.68 ± 9.05 mmHg at baseline to 52.28 ± 10.97 mmHg on Day 7), which was not clinically significant. Pulse rate declined from 111.22 ± 12.51 bpm to 108.58 ± 11.61 bpm, while respiratory rate decreased from 27.92 ± 3.35 breaths/min to 26.53 ± 3.18 breaths/min from baseline to Day 7, both consistent with fever resolution.

Safety evaluation

The safety of mefenamic acid oral suspension was systematically evaluated using hematological, hepatic, renal, and urinary parameters, assessed at baseline (Day 1) and post-treatment (Day 7) in a cohort of 50 pediatric subjects (Table 2). No treatment-related adverse events were observed. The findings demonstrated that the intervention was well tolerated, with no clinically significant or treatment-emergent laboratory abnormalities.

**Table 2:** Summary of Hematological and Biochemical Parameters Values expressed as mean ± SD. A t-test p-value < 0.05 was considered statistically significant. BUN: Blood Urea Nitrogen; Hb: Hemoglobin; MCH: Mean Corpuscular Hemoglobin; MCHC: Mean Corpuscular Hemoglobin Concentration; MCV: Mean Corpuscular Volume; RBC: Red Blood Cell; SGOT: Serum Glutamic Oxaloacetic Transaminase; SGPT: Serum Glutamic Pyruvic Transaminase; TLC: Total Leukocyte Count

Examination	Parameters	Baseline Visit	Day 7	t-value	p-value
Complete Blood Count	Hb (g/dL)	11.22 ± 1.28	11.33 ± 1.33	0.42	0.674
	TLC (10^9^/L)	9343.60 ± 3790.65	8859.40 ± 2826.40	-0.72	0.471
	Neutrophil (%)	51.54 ± 17.16	45.28 ± 15.96	-1.89	0.062
	Lymphocytes (%)	40.00 ± 17.71	44.98 ± 17.33	1.42	0.158
	Basophil (%)	0.02 ± 0.14	0.00 ± 0.00	-1.01	-
	Eosinophil (%)	1.90 ± 0.94	2.10 ± 1.36	0.86	0.394
	Hematocrit (%)	36.29 ± 5.44	37.29 ± 7.69	0.75	0.455
	Total RBC count (10^12^/L)	4.85 ± 0.52	4.67 ± 0.56	-1.67	0.099
	RBC dispersion width (%)	15.45 ± 2.54	19.09 ± 27.37	0.94	0.351
	Platelet count (10^9^/L)	329000.00 ± 91373.30	347987.22 ± 120544.75	0.89	0.377
	MCH (pg)	23.27 ± 3.71	24.85 ± 4.06	2.03	0.045
	MCHC (g/dL)	31.36 ± 2.98	31.80 ± 3.05	0.73	0.467
	MCV (fL)	74.54 ± 11.98	78.39 ± 12.12	1.60	0.113
Liver Function Test	Alkaline phosphatase (U/L)	193.37 ± 88.36	174.52 ± 65.46	-1.21	0.228
	Bilirubin - direct (mg/dL)	0.18 ± 0.09	0.17 ± 0.11	-0.50	0.620
	Bilirubin - indirect (mg/dL)	0.26 ± 0.22	0.26 ± 0.28	0.00	1.000
	Serum bilirubin - total (mg/dL)	0.44 ± 0.31	0.44 ± 0.37	0.00	1.000
	SGOT (U/L)	28.67 ± 9.80	27.80 ± 9.96	-0.44	0.661
	SGPT (U/L)	16.08 ± 6.21	15.95 ± 7.90	-0.09	0.927
Kidney Function Test	BUN (mg/dL)	18.92 ± 6.60	19.61 ± 6.26	0.54	0.593
	Serum creatinine (mg/dL)	0.42 ± 0.14	0.43 ± 0.14	0.36	0.722

Hematological parameters remained largely within normal physiological limits across the study duration. No statistically significant changes were observed in hemoglobin (p = 0.6744), total leukocyte count (p = 0.4707), platelet count (p = 0.3769), or red blood cell indices, including mean corpuscular volume (MCV) (p = 0.1134), mean corpuscular hemoglobin concentration (MCHC) (p = 0.4674), and red cell distribution width (RDW) (p = 0.3514). Lymphocyte and neutrophil percentages demonstrated mild fluctuations (p = 0.1584 and p = 0.0619, respectively), though not reaching statistical significance. A statistically significant increase was noted in mean corpuscular hemoglobin (MCH), from 23.27 ± 3.71 to 24.85 ± 4.06 pg (p = 0.0449); however, this variation remained within normal reference ranges and was not clinically relevant. Basophil counts declined uniformly to 0.00 ± 0.00 on Day 7, precluding comparative statistical analysis.

Biochemical analyses indicated no hepatic or renal toxicity. Liver enzymes, including serum glutamic oxaloacetic transaminase (SGOT) (p = 0.6607), serum glutamic pyruvic transaminase (SGPT) (p = 0.9273), and alkaline phosphatase (p = 0.2284), showed non-significant changes. Bilirubin values (total, direct, and indirect) remained stable (p > 0.6) and within physiological ranges. Renal function markers, including blood urea nitrogen (BUN) (p = 0.5929) and serum creatinine (p = 0.7218), also exhibited no statistically or clinically significant changes. Urinalysis revealed no adverse changes in urinary pH (p = 0.3508), with physical characteristics - appearance and color - remaining within expected norms. Trace hematuria was observed in two cases, without associated clinical symptoms.

Assessment of GI tolerability and FOBT

The GI safety of mefenamic acid oral suspension was evaluated using a structured symptom-based questionnaire and FOBT. Throughout the study, none of the 50 pediatric participants reported GI symptoms, such as abdominal pain, bloating, nausea, vomiting, heartburn, dysphagia, or altered bowel habits, indicating excellent GI tolerability. The two subjects with trace FOBT positivity showed transient findings on Day 3 that resolved by Day 7, with repeat tests returning negative and no associated GI symptoms or clinically significant abnormalities on assessment. These isolated findings were not considered indicative of GI mucosal injury or bleeding.

## Discussion

This prospective observational study suggests that mefenamic acid oral suspension was associated with a rapid and sustained reduction in body temperature in pediatric patients with febrile illness, along with a favorable short-term safety and tolerability profile. Most children achieved normothermia within the first few doses, and fever resolution was maintained throughout the treatment and follow-up period, without treatment-related adverse events.

Fever in children can result from infections, inflammatory conditions, or systemic illnesses, requiring prompt and effective antipyretic management. Studies have shown that children with febrile seizures exhibit significantly higher levels of NLRP3 and IL-1β compared to febrile controls. Activation of the NLRP3 inflammasome promotes IL-1β maturation and release, which can increase neuronal excitability and contribute to seizure activity [10,11]. Given the involvement of NLRP3 and IL-1β in febrile seizures, targeting these pathways may offer therapeutic benefits. Mefenamic acid exerts its therapeutic effects through a dual mechanism of action: inhibition of COX enzymes, leading to reduced PGE₂ synthesis, and suppression of the NLRP3 inflammasome, resulting in decreased production of proinflammatory cytokines. This combined activity supports its clinical utility as a reliable option for fever management and may offer additional benefits in lowering the risk of febrile seizures in pediatric patients [12,13].

The antipyretic efficacy of mefenamic acid in children has been well-documented in previous studies. Reddy et al. (2020) reported that mefenamic acid demonstrated significantly greater fever reduction compared to both paracetamol and ibuprofen in febrile children, with a more pronounced and sustained effect during the two- to four-hour observation period [14]. In our study, mefenamic acid oral suspension exhibited a rapid onset of antipyretic action, with most pediatric participants recording reduced temperature after the initial dose. This effective action continued over the course of treatment, with a consistent reduction in body temperature observed by Day 3 evening. These findings are consistent with the established pharmacokinetic profile of mefenamic acid [15] and are further supported by Khubchandani et al. (1995) [16], who reported a sustained antipyretic response and superior tolerability compared to other commonly used antipyretics. The structured assessment of fever-free intervals across three daily timepoints provided an objective measure of fever control, demonstrating a progressive increase in afebrile periods, culminating in complete resolution by Day 4. This sustained fever control contributes to improved patient comfort, reduced night-time disturbances, and greater convenience for caregivers. Collectively, these results reinforce the clinical utility of mefenamic acid as a reliable antipyretic option in pediatric practice.

Concerns have been raised regarding the safety of NSAIDs in children, particularly the potential risks of hematuria and GI complications when used concomitantly with antibiotics or corticosteroids [17,18]. However, findings from the present study demonstrated no such adverse effects associated with mefenamic acid oral suspension. Urinalysis and fecal occult blood testing showed no abnormalities, and GI tolerability was confirmed through patient-reported outcomes and symptom diaries, with no reports of abdominal pain, vomiting, or diarrhea. Hematological and biochemical parameters assessing hepatic and renal function remained within normal limits, indicating that mefenamic acid, when administered at the recommended optimal dose of 5 mg/kg per dose, three times daily, is generally well tolerated in pediatric patients. These observations are consistent with the recent consensus by Pai et al. (2025) [6], which emphasized that clinical studies in children have demonstrated mefenamic acid’s favorable safety and tolerability profile, with minimal adverse effects and no significant GI, hepatic, or renal toxicity when used appropriately. Overall, the findings support the use of mefenamic acid oral suspension as a reliable and well-tolerated antipyretic agent in pediatric practice.

In current pediatric fever management, antipyretic therapy primarily aims to improve comfort, with antipyretics commonly used as first-line agents. In routine practice, alternative NSAIDs, such as mefenamic acid, are often considered when longer-lasting antipyretic effects or additional analgesic and anti-inflammatory benefits are desired. The present real-world findings suggest that mefenamic acid oral suspension may be a practical option in selected pediatric patients, providing sustained fever control with good short-term tolerability.

Strengths and limitations of the study

The strengths of this study include its real-world design, which closely reflects routine pediatric clinical practice, and the comprehensive evaluation of both efficacy and safety parameters. Additionally, the inclusion of a broad pediatric age range and the assessment of patients receiving concomitant therapies enhance the external validity and applicability of the findings. However, certain limitations of the study should be acknowledged. The uncontrolled, open-label study design does not allow causal conclusions and cannot distinguish treatment effects from the natural resolution of fever. The use of concomitant medications may have influenced outcomes. In addition, the single-center design, small sample size, and short follow-up period limit the generalizability of the outcomes. Accordingly, the results should be considered descriptive and hypothesis-generating, and larger, multicenter, randomized controlled trials are needed to confirm comparative efficacy and safety in diverse pediatric populations.

## Conclusions

The present observational study suggests that mefenamic acid oral suspension is associated with a rapid onset and sustained reduction of fever in pediatric patients, along with improvement in associated clinical symptoms. Short-term safety assessments demonstrated no clinically relevant alterations in hematological or biochemical parameters, indicating a favorable tolerability profile when used at recommended doses, including in children receiving concomitant medications.

## Figures and Tables

**Figure 3:**
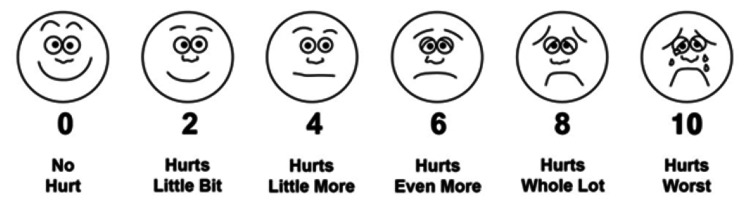
Face Pain Rating Scale

## Appendices

Appendix 1: Gastrointestinal tolerability questionnaire

Did the subject experience the following symptoms during the last week

**Table 3:** Gastrointestinal Tolerability Questionnaire

S. No.	Symptom	None	Mild	Moderate	Quite a lot	Severe		Very severe	Unbearable
										1	Abdominal pain								
In common										
Postprandial										
Fasting										
Doesn't decline after defecation										
									2		Epigastric pain								
In common									
During daytime									
At night/ asleep									
3	Bloating								
4	Regurgitation								
5	Heartburn								
6	Belching								
7	Abdominal rumbling								
										8	Loss of appetite								
9	Empty feeling								
10	Nausea								
11	Vomiting								
12	Postprandial fullness								
									13	Flatulence								
14	Haematemesis								
15	Dysphagia								
	Liquid food								
	Solid food								
16	Stools								
	Melena								
	Bloody								
	Mucous								
	Frequent hard								
	Frequent loose								
	Alternating solid or loose								
									17	Constipation								
Frequently with pain									
								Urging stools									
Strong pressing									
								Steatorrhea									

18) Describe the subject’s abdominal or epigastric pain during the last week

Face Pain Rating Scale
